# *PLA2G6*-associated neurodegeneration (PLAN): Further expansion of the clinical, radiological and mutation spectrum associated with infantile and atypical childhood-onset disease

**DOI:** 10.1016/j.ymgme.2014.03.008

**Published:** 2014-06

**Authors:** M.A. Illingworth, E. Meyer, W.K. Chong, A.Y. Manzur, L.J. Carr, R. Younis, C. Hardy, F. McDonald, A.M. Childs, B. Stewart, D. Warren, R. Kneen, M.D. King, S.J. Hayflick, M.A. Kurian

**Affiliations:** a Department of Neurology, Great Ormond Street Hospital, London, UK; b Neurosciences Unit, UCL-Institute of Child Health, London, UK; c Department of Radiology, Great Ormond Street Hospital, London, UK; d Dubowitz Neuromuscular Centre for Congenital Muscular Dystrophies and Myopathies, Great Ormond Street Hospital, London, UK; e West Midlands Regional Genetics, Birmingham Women's Hospital, Birmingham, UK; f Department of Paediatric Neurology, Leeds General Infirmary, Leeds, UK; g Department of Paediatrics, York Teaching Hospitals NHS Foundation Trust, York, UK; h Department of Neuroradiology, Leeds teaching Hospitals, Leeds. UK; i Department of Neurology, Alder Hey Children's Hospital, Liverpool, UK; j Department of Paediatric Neurology, Children's University Hospital, Temple Street, Dublin, Ireland; k Department of Molecular & Medical Genetics, OR Health & Science University, Portland 97239, USA; l Department of Paediatrics, OR Health & Science University, Portland 97239, USA; m Department of Neurology, OR Health & Science University, Portland 97239, USA

**Keywords:** Neurodegeneration with brain iron accumulation, NBIA, *PLA2G6*, INAD, PLAN

## Abstract

Phospholipase A2 associated neurodegeneration (PLAN) is a major phenotype of autosomal recessive Neurodegeneration with Brain Iron Accumulation (NBIA). We describe the clinical phenotypes, neuroimaging features and *PLA2G6* mutations in 5 children, of whom 4 presented with infantile neuroaxonal dystrophy (INAD). One other patient was diagnosed with the onset of PLAN in childhood, and our report highlights the diagnostic challenges associated with this atypical PLAN subtype. In this series, the neuroradiological relevance of classical PLAN features as well as apparent claval hypertrophy’ is explored. Novel *PLA2G6* mutations were identified in all patients. PLAN should be considered not only in patients presenting with a classic INAD phenotype but also in older patients presenting later in childhood with non-specific progressive neurological features including social communication difficulties, gait disturbance, dyspraxia, neuropsychiatric symptoms and extrapyramidal motor features.

## 1 Introduction

‘Neurodegeneration with Brain Iron Accumulation’ (NBIA) encompasses a group of disorders characterised by progressive motor symptoms, neurological regression and radiologically discernible brain iron accumulation [1–4]. The major childhood NBIA syndromes include pantothenate kinase associated neurodegeneration (PKAN; MIM#234200) [5], fatty acid hydroxylase associated neurodegeneration (FAHN; MIM#612319) [6], mitochondrial membrane protein associated neurodegeneration (MPAN; MIM#614298) [7], beta-propeller protein associated neurodegeneration (BPAN; MIM#300894) [8], and phospholipase A_2_ associated neurodegeneration (PLAN; MIM#256600). PLAN is an autosomal recessive disorder caused by mutations in the ubiquitously expressed *PLA2G6* gene (MIM*603604) [9]. *PLA2G6* encodes the 85 kDa protein, iPLA_2_-VI which has key functions in maintaining cell membrane homeostasis, through phospholipid remodelling, regulation of apoptosis and catalysing the hydrolysis of glycerophospholipids [2,4,6,10–13]. Features of the human disease are recapitulated in the *PLA2G6* knockout mouse [14] with axonal spheroid formation secondary to deranged cell membrane homeostasis leading to the accumulation of membrane phospholipids and mitochondrial degeneration. *PLA2G6*-associated disease is a continuum of three distinct, yet overlapping phenotypes [6,10]: classic infantile neuroaxonal dystrophy (INAD) (MIM#256600), atypical neuroaxonal dystrophy (NAD) of childhood-onset (MIM#610217) and *PLA2G6-*related dystonia-parkinsonism with onset in adulthood (MIM#612953) [1,6]. These phenotypes are pathologically characterised by the presence of axonal spheroids and progressive brain iron deposition and clinically by progressive motor dysfunction and cognitive decline. We report 5 new cases of PLAN and describe both infantile and atypical childhood-onset phenotypes with novel radiological features and previously unreported *PLA2G6* mutations.

## 2 Methods

Patients with clinical and radiological features suggestive of PLAN were identified from tertiary neurology services in the UK and Ireland. Recruiting paediatric neurology centres included Great Ormond Street Hospital, Leeds General Infirmary, Alder Hey Children's Hospital and the Children's University Hospital Dublin. The patient medical notes were reviewed to ascertain relevant clinical history and results of investigations. Where available, video footage of the affected children was also examined. All available MR neuroimaging was reviewed by a paediatric neuroradiologist. For most patients, *PLA2G6* screening was undertaken in the diagnostic setting by the West Midlands Regional Genetic Service, Birmingham, UK. Direct Sanger sequencing of the coding regions and flanking intronic regions was undertaken for all patients [15]. Additional multiplex ligation probe amplification (MLPA) analysis for Patient 4 was also performed using previously described primers and techniques [15].

## 3 Clinical cases

### 3.1 Case 1

A female infant, born to non-consanguineous Caucasian parents, presented at 15 months of age with global developmental regression. Early development had been age appropriate until 12 months of age. Her development thereafter plateaued, with regression from 14 months. She lost all previously acquired vocabulary, fine motor skills and the ability to sit. Choking during feeds and excess drooling suggested the onset of bulbar dysfunction. She was non-dysmorphic. Axial tone was reduced with peripheral hypertonicity and hyperreflexia. Plantar responses were down-going. Ophthalmological examination was normal. A rapid clinical decline ensued. Deteriorating bulbar function necessitated gastrostomy tube feeding at 20 months. At 26 months of age spontaneous movement was minimal with arreflexia (Table 1).

**Fig. 1:**
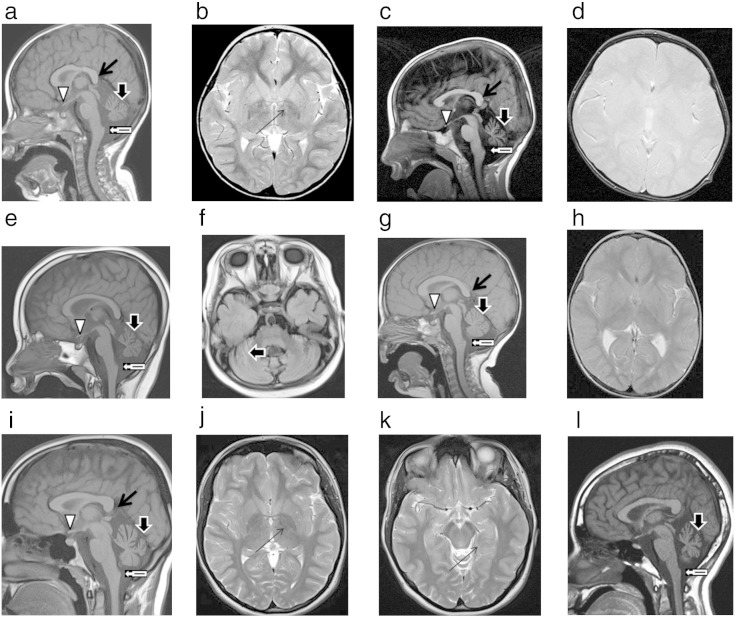
a. Midline sagittal T1 brain MRI of Case 1 at 27 months. b. Axial T2 weighted brain MRI of Case 1 aged 39 months, demonstrating iron deposition in the globus pallidus, which is absent on earlier imaging (a). c. Midline sagittal T1 brain MRI of Case 2 aged 33 months, demonstrating classical features of typical PLAN (symbol key and Table 3) and apparent claval hypertrophy. d. Axial T2* brain MRI of Case 2 aged 33 months, at the level of the globus pallidus; note the absence of iron deposition. e. Midline sagittal T1 brain MRI of Case 3 aged 4 years and 6 months, demonstrating classical features of typical PLAN and apparent claval hypertrophy. f. Axial FLAIR brain MRI of Case 3 aged 4 years and 6 months at level of cerebellum, showing high signal of the cerebellar cortex, indicative of gliosis. There is no iron deposition. g. Midline sagittal T1 brain MRI of Case 4 aged 22 months, demonstrating cerebellar atrophy, apparent claval hypertrophy and thin smooth splenium of the corpus callosum. h. Axial T2 weighted brain MRI of Case 4 aged 22 months, at the level of the globus pallidus. There is no iron deposition. i. Midline sagittal T1 brain MRI of Case 5 aged 11 years, demonstrating cerebellar atrophy and apparent claval hypertrophy. j. Axial T2 brain MRI of Case 5 aged 16 years demonstrating iron deposition in the globus pallidus. k. Axial T2 brain MRI of Case 5 aged 16 years demonstrating iron deposition in the substantia nigra. l. Midline sagittal T1 brain MRI of a 10 year old female with hemiplegic migraine and pathogenic *CACNA1A* mutation, demonstrating cerebellar atrophy and apparent claval hypertrophy. **Table:** 

Electroencephalogram (EEG) showed diffuse slowing of background activity with infrequent and brief paroxysms of bi-posterior sharp theta and spike-slow wave activity, without clinical correlation (Table 2). Electromyogram (EMG) demonstrated changes consistent with chronic denervation. Initial MRI brain scan (without T2* sequences) at 27 months of age showed a hypoplastic cerebellum, small pons and vertically orientated smooth splenium of the corpus callosum. There was evidence of visual pathway atrophy and a small optic chiasm, with white matter signal intensity, which may represent delayed myelination. There was no evidence of brain iron deposition. Subsequent imaging at 39 months with T2* sequences demonstrated iron deposition in the globus pallidus, indicated by hypointensity on T2* sequences. The cerebellar hypoplasia was non-progressive, with apparent claval prominence (Table 2, Fig. 1a and b).

### 3.2 Case 2

A male infant, the first child born to consanguineous first cousin Caucasian parents, presented at 12 months of age with developmental arrest. Preceding history was unremarkable and acquisition of developmental milestones in the first year was age appropriate. He failed to acquire skills thereafter and regressed from 15 months of age. He was non-dysmorphic with axial hypotonia and hypertonicity of all 4 limbs. Rapid neurocognitive decline ensued and by 15 months of age. Nystagmus and strabismus were evident with evolving four limb spasticity and distal limb arreflexia. Ophthalmological examination at 18 months confirmed optic atrophy. Bulbar dysfunction was suggested by significant drooling and gastrostomy feeding commenced at 24 months (Table 1).

**Fig. 2:**
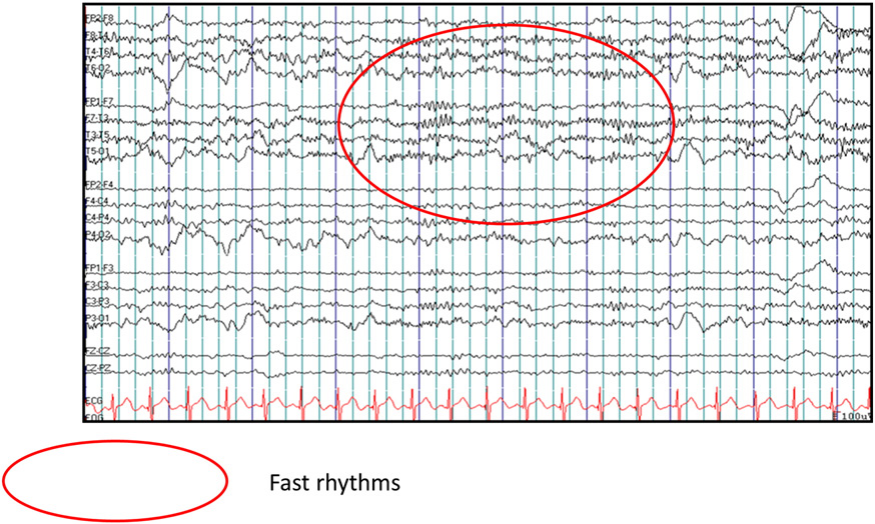
Electroencephalogram, Case 2 EEG of Case 2, aged 29 months in wakefulness, demonstrating fast beta2 activity (indicated by the red oval shape).

EEG at 30 months of age shows high amplitude fast activity (Table 2, Fig. 2). Neurophysiology was consistent with a sensorimotor axonal neuropathy. MRI brain scan at 29 months (Table 2, Fig. 1c and d) showed advanced cerebellar and pontine atrophy. A smooth, thin vertically orientated splenium of the corpus callosum was present. Myelination was delayed and the optic tracts underdeveloped with a small optic chiasm. High signal in the cerebellar grey matter was consistent with gliosis. Again, there was apparent claval prominence, without evidence of brain iron deposition.

### 3.3 Case 3

Case 3 presented at 18 months of age with motor regression. She is the only child of consanguineous Pakistani parents. She was born following an uncomplicated pregnancy and attained early developmental milestones age appropriately. Following a febrile illness at 7 months of age, her rate of acquisition of developmental milestones slowed. Regression from 18 months of age manifested as progressive weakness, loss of independent ambulation and ‘unsteadiness’. By 24 months, she had lost expressive language and at 5.5 years she can no longer sit independently, has no purposeful hand movement and requires a modified pureed diet due to bulbar dysfunction. A gastrostomy is planned due to frequent choking episodes. Paroxysmal events emerged at 2.5 years, involving tonic posturing and stiffening without electrographic correlate. On examination, she has bilateral horizontal nystagmus and optic atrophy but no strabismus. She is hypotonic axially with peripheral hypertonicity, and contractures are evident at the knees and ankles with arreflexia. Her vision has deteriorated over time and now, aged 5.5 years she has no functional vision. Secretions and excessive drooling are managed with transdermal hyoscine patches. Omeprazole, gavison and erythromycin are used in the management of her gastro-oesophageal reflux. Minimal benefit is observed from the use of baclofen for tone management. Lower limb plantar flexion contractures are managed with orthotics, and she has been referred for bilateral tendon releases.

Initial EEG (2.5 years) demonstrates diffuse beta fast activity and bilateral independent spike discharges, with anterior predominance. MRI brain scan at 4.5 years (Fig. 1e and f) shows cerebellar hypoplasia and T2 cerebellar high signal consistent with gliosis, underdevelopment of the optic tracts with a small optic chiasm. There is a generalised lack of cerebral white matter bulk and apparent claval prominence.

### 3.4 Case 4

Case 4 is male, the first child born to his non-consanguineous, white Caucasian parents. Antenatal, birth and family history is unremarkable. He presented with global developmental regression from 22 months, and preceding developmental milestones were attained age appropriately. Prior to presentation he used some single words appropriately, cruised around furniture and had a functional bimanual pincer grip. A convergent strabismus was apparent from 14 months which was managed with patching, though over the subsequent months optic atrophy and visual deterioration ensued. Regression at 22 months manifested as frequent falls, loss of the ability to stand independently, reduced crawling and loss of a pincer grip. He was no longer able to hold or drink from a two handled beaker. Prolonged feeding times and an increased frequency of choking were suggestive of evolving bulbar dysfunction. He currently experiences paroxysmal events, with a history and semiology indicative of breath holding spells. Recently nocturnal unprovoked apnoeic episodes have emerged and overnight polysomnography implies a centrally mediated mechanism. Examination at 24 months of age, demonstrated axial hypotonia, profuse drooling and an open mouthed posture, suggestive of lower facial weakness. He had a convergent squint but full range of eye movements without nystagmus. Power was at least antigravity and there was no increase in dynamic tone or dystonia. Reflexes were brisk and plantar responses were upgoing. He had no overt cerebellar features. He remains alive at 30 months of age.

EEG at 24 months demonstrated excess fast activity. Changes consistent with motor neuronopathy peripherally and of genioglossus were detected on electromyogram (EMG). Whilst the electroretinogram (ERG) was normal, visual evoked responses (VERs) were reduced. A respiratory sleep study, undertaken in view of unprovoked apnoea at night demonstrated central apnoeas at 26 months of age, and subsequently, he remains under the care of the respiratory physicians. A videofluorscopy is planned.

### 3.5 Case 5

This female child was born to non-consanguineous Caucasian parents in good condition at 41 week gestation by ventouse extraction. Apart from some minor bleeding, the pregnancy was uneventful. Early development was reassuring, with acquisition of gross motor, fine motor and speech and language skills age appropriately. Concerns were first expressed at 3 years, when she developed an unsteady, broad based gait and she experienced some difficulty in settling in the nursery school environment. Her gait remained ataxic, but stable without further regression of skills and she commenced mainstream education. She then presented at 10 years of age with a 12 month history of further gait deterioration, with poor balance, bilateral pes cavus deformity and left talipes equinovarus. Examination at 11 years demonstrated brisk upper limb reflexes and absent lower limb reflexes with equivocal plantar responses. A mild scoliosis was noted, which is managed conservatively. Opthalmological assessment at 11 years confirmed hypometric eye saccades without nystagmus or strabismus. Features evolved over time, with deterioration in independent mobility secondary to increasing ataxia and evolution of dystonic limb movements. At 16 years she acquired a rollator to facilitate independence. Her speech is preserved, though intermittent dysarthria is reported. At present she tolerates a normal enteral diet; however, mealtimes are becoming prolonged and occasional swallowing difficulty is reported.

A clinical psychology assessment at 14 years demonstrated verbal and non-verbal IQs within the low-average range and confirmed parental concerns regarding poor working memory and word finding difficulties. She is described as ‘short-tempered’, ‘rigid’, ‘obsessional’ and ‘routine-orientated’. To date, treatment has been symptomatic. Achilles tendon lengthening was undertaken at 11 years, without sustained benefit. Her mild scoliosis is monitored annually and at present she does not require bracing or surgery. To date there have been no trials of antispasmodic/dystonic agents.

EMG and NCS showed changes consistent with chronic denervation. In light of these findings, a muscle biopsy was undertaken. Muscle histopathology (Table 2) showed neurogenic changes and cytochrome oxidase activity (COX, complex IV) was reduced at 0.007 (reference 0.014–0.034) on respiratory chain enzyme (RCE) analysis.

Initial MRI brain scan undertaken at 11 years of age showed cerebellar hypoplasia and evidence of early iron deposition. Subsequent imaging at 16 years showed progressive cerebellar atrophy and brain iron deposition in the substantia nigra and globus pallidus. (Table 2, Fig. 1k and l).

## 4 Molecular genetic investigations

All patients had either homozygous or compound heterozygous mutations in the *PLA2G6* gene (Table 3). Novel mutations were identified in all patients including deletions, duplications, missense and nonsense mutations.

## 5 Discussion

We report the clinical findings and neuroimaging features of 5 children (4 with phenotypes consistent with classic INAD and 1 with atypical NAD) in whom novel *PLA2G6* mutations were identified. *PLA2G6* mutations account overall for approximately 20% of childhood onset NBIA [6]. Whilst the exact prevalence is unknown, it is estimated at 1:1,000,000 [6]. Infantile-onset INAD is the most common, with a relatively homogenous phenotype [1,6]. This is clearly illustrated in our case series, all typical INAD patients presented prior to their third birthday, with neurodevelopmental regression, progressive 4 limb spasticity, bulbar dysfunction and denervation on nerve conduction studies. In keeping with published data, strabismus, optic atrophy, and fast rhythms on EEG are commonly, but not universally reported [3]. Even within this relatively homogenous group, a degree of phenotypic variability is often reported, as evident in Patients 1 and 2 who appeared to have an arguably more aggressive disease course than most reported patients with typical PLAN.

In contrast to INAD, the onset of atypical NAD is usually outside the infantile period in early childhood (1.5–4.4 years) [4]. Atypical NAD is less common than INAD, and the phenotype is more heterogeneous rendering the diagnosis challenging. Autistic features with disordered social communication and interaction may predominate initially, with gait disturbance, tetraparesis, extrapyramidal features and cerebellar signs evolving over decades [1,4]. In atypical NAD difficult neuropsychiatric features may evolve [1,4], a feature also observed in the third NBIA, *PLA2G6* phenotype-PARK14-linked parkinsonism [10,16,17]. In patients with PARK14 early onset dystonia-parkinsonism (< 30 years), *PLA2G6* mutations may be identified in up to 6.9%. Typical presentation is in adolescence, with the onset of parkinsonism [6,10,16]. Cognitive decline and neuropsychiatric features thereafter predominate. In contrast with infantile and childhood atypical NAD, specific neuroradiological changes are minimal and in many cases only generalised cerebral atrophy is demonstrated; frontotemporal atrophy with hypoperfusion on SPECT may occur. Brain iron accumulation occurs late and is relatively rare [6,10,16].

This report highlights the degree of phenotypic overlap with atypical NAD and the neuropsychiatric features of adult NBIA phenotypes. Patient 5 who is described as ‘short-tempered’, ‘rigid’, ‘obsessional’ and ‘routine-orientated’ presented with a gradual onset of gait disturbance and speech regression. Her clinical deterioration was insidious and manifested as a slowly evolving movement disorder. At 16 years of age she is ambulant, intermittently dysarthric and remains in mainstream education.

Our case series highlights the classical neuroimaging features of PLAN. Cerebellar hypoplasia and T2-weighted high signal in the cerebellum (suggesting cerebellar gliosis), hypoplastic optic tracts/chiasm and an elongated, vertically orientated splenium were seen in all typical INAD cases reported here. Consistent with published data, iron deposition was not detected on initial neuroimaging in any of the infantile cases (neuroimaging undertaken performed at 22–29 months) but became evident in Case 1 on repeat imaging following a 12 month interval. ‘Apparent claval hypertrophy’ with pontine atrophy has been proposed as an early radiological marker of typical PLAN with the presence of prominent spheroid bodies in the claval nuclei on histopathology [18]. In this reported series, apparent claval hypertrophy was evident in all typical and atypical cases of PLAN. However, we have observed this radiological feature in other disorders associated with cerebellar atrophy (Fig. 1l, Supplementary Fig. 1). Hence, we postulate that the claval hypertrophy is not specific for PLAN and is merely another radiological feature aiding diagnosis but not pathognomonic for PLAN, a likely epiphenomenon, reflecting adjacent pontocerebellar atrophy as opposed to true hypertrophy of medullary structures.

Our case series highlights the classical neuroimaging features of PLAN. Cerebellar hypoplasia and T2-weighted high signal in the cerebellum (suggesting cerebellar gliosis), hypoplastic optic tracts/chiasm and an elongated, vertically orientated splenium were seen in all typical INAD cases reported here. Consistent with published data, iron deposition was not detected on initial neuroimaging in any of the infantile cases (neuroimaging undertaken performed at 22–29 months) but became evident in Case 1 on repeat imaging following a 12 month interval. ‘Apparent claval hypertrophy’ with pontine atrophy has been proposed as an early radiological marker of typical PLAN with the presence of prominent spheroid bodies in the claval nuclei on histopathology [18]. In this reported series, apparent claval hypertrophy was evident in all typical and atypical cases of PLAN. However, we have observed this radiological feature in other disorders associated with cerebellar atrophy (Fig. 1l, Supplementary Fig. 1). Hence, we postulate that the claval hypertrophy is not specific for PLAN and is merely another radiological feature aiding diagnosis but not pathognomonic for PLAN, a likely epiphenomenon, reflecting adjacent pontocerebellar atrophy as opposed to true hypertrophy of medullary structures.

The novel *PLA2G6* mutations reported in this series contribute to the expanding spectrum of variants associated with PLAN. Whilst the c.1634A > C, p.Lys545Thr homozygous mutation is common in some Pakistani families [3], mutation heterogeneity is common with no evidence of mutation hotspots. Current genetic sequencing methods (Sanger sequencing and MLPA) detect a predicted 85%–90% of pathogenic mutations. The remainder may represent intronic mutations and promoter variants, unidentifiable by current diagnostic sequencing methods [4,17]. Genotype–phenotype correlation in PLAN is not strikingly obvious. Nevertheless, it has been postulated that those with two null mutations tend to manifest the severe INAD phenotype whilst compound heterozygotes for missense variants present later, with atypical NAD [2]. We however report the unusual finding of a patient with atypical NAD (Case 5) and the mildest phenotype of all our cases, who is compound heterozygous for a novel missense mutation and null mutation. In addition to allelic heterogeneity, it is likely that other, currently undetermined genetic, epigenetic and environmental factors contribute to phenotypic presentation.

*PLA2G6* encodes the 85 kDa calcium-independent group VIA phospholipase A_2_ enzyme, iPLA2β. Proposed pathogenic mechanisms relate to its role in maintaining cell membrane homeostasis [24]. Recently, calcium dysregulation in astroglial networks has been described in mutant mouse models, providing further insight into disease pathogenesis [13]. Mitochondrial, axon and dendrite membrane dynamics may be aberrantly affected by disrupted iPLA2β. Axonal spheroids, the histopathological hallmark of *PLA2G6-*associated disease, represent the degeneration of mitochondrial inner membranes [20,21,22]. Whether this disruption of mitochondrial architecture affects respiratory chain function is unknown, as mitochondrial investigations are not commonly undertaken in PLAN. The insidious onset of disease in Patient 5 warranted detailed neurometabolic investigation, including muscle biopsy which was undertaken at 11 years. Muscle respiratory chain enzyme analysis revealed severely reduced cytochrome oxidase (COX) activity. Of the respiratory chain enzymes, cytochrome oxidase is particularly vulnerable to oxidative stress [22] and calcium dysregulation in mitochondria and astrocytes results in oxidative damage in the mouse model [13]. Normally functioning VIA-iPLA2 protects mitochondria from oxidative stress and valid mitochondria are necessary to meet the high energy demands of neuronal function. Degeneration of mitochondrial inner membranes has been observed in the *PLA2G6* knock-out mouse [19]. It is possible therefore that the low COX activity measured in Case 5 may be secondary to oxidative stress due to impaired VIA-iPLA2, since secondary derangement of respiratory chain enzymes is not reported in neurogenic muscle. There is one other atypical PLAN case reported in the literature in whom respiratory chain complexes were measured, demonstrating global deficiency of all complexes on muscle biopsy at 12 years of age, in the presence of reduced mitochondrial density [23]. Mitochondrial degeneration releases stress-inducers including reactive oxygen species, cytochrome c and lipid peroxisomes, resulting in cumulative mitochondrial degeneration with the absence of normally protective VIA-iPLA2 contributing to further damage [19]. We postulate that such secondary respiratory chain enzyme deficiencies in PLAN may progress over time, and the impact of oxidative damage to the mitochondria secondary to calcium dysregulation may be cumulative, paralleling clinical decline. Indeed, the impact of *PLA2G6* mutations on the mitochondrion and respiratory chain requires further evaluation, and whether anti-oxidant therapy may have a protective role in preventing secondary respiratory chain dysfunction [2, 24].

PLAN is relentless and irreversible, and currently no disease modifying treatments exist. For infantile onset PLAN, disease course is the most progressive with rapidly ensuing spasticity, contractures, cognitive decline in childhood, and death often reported at the end of the 1st decade. Atypical NAD seems to have a less severe disease course, and the rarity of adult onset PLAN precludes prognostication, although some adults with this form of disease do show rapid cognitive decline from disease onset in early adulthood. As our case series highlights, treatment strategies focus on a multidisciplinary approach to optimise nutrition, prevent cardiorespiratory complications and maintain orthopaedic vigilance [1]. Pharmacological agents are often used to manage symptoms such as gastro-oesophageal reflux (anti-reflux agents), excess secretions (glycopyrrolate, hyoscine) and dystonia/spasticity (trihexyphenidyl/baclofen) [1]. As we have discussed, contracture management can be challenging, requiring not only orthotics but also more invasive strategies such as botulinum toxin and tendon lengthening. As the TIRCON (**T**reat **Ir**on **R**elated **C**hildhood **O**nset **N**eurodegeneration FP7 277984-2) trial evaluates the potential efficacy of the iron chelator, deferiprone in PKAN patients, there is optimism for its extrapolation to other NBIA disorders, including PLAN, should it be beneficial. Further unravelling of the function of *PLA2G6* and interacting pathways will be fundamental to development of more rational and specific molecular-based therapeutic strategies.

The following are the supplementary data related to this article

Supplementary Fig. 1 9 images of midline sagittal T1 MRI brain imaging, indicating the presence of apparent claval hypertrophy (indicated by yellow arrow) in all cases.
**1a** Section of midline sagittal T1 MRI brain scan, at the level of the cerebral aqueduct, of a child aged 2 years 1 month with *CASK* mutation, cerebellar hypoplasia and apparent claval hypertrophy.
**1b** Section of midline sagittal T1 MRI brain scan at the level of the cerebral aqueduct, of a female aged 3 years 4 months, with pontocerebellar hypoplasia of undetermined cause. The section shows apparent claval hypertrophy and cerebellar hypoplasia.
**1c** Section of midline sagittal T1 MRI brain scan, at the level of the cerebral aqueduct, of a female aged 3 years and 10 months, with pontocerebellar hypoplasia type 2 secondary to *TSEN54* mutation. The section capturing pontocerebellar structures, demonstrates cerebellar hypoplasia and apparent claval hypertrophy.
**1d** Section of midline sagittal T1 MRI brain scan, at the level of the cerebral aqueduct of a 9 years 7 month year old male, with epilepsy, learning and behavioural difficulties of undetermined cause. The section, highlighting pontocerebellar structures demonstrates cerebellar hypoplasia and apparent claval hypertrophy.
**1e** Section of midline sagittal T1 MRI brain scan of a 6 years and 10 month old male with a four limb movement disorder, epilepsy, microcephaly and learning difficulties of undetermined cause. *PLA2G6* mutation testing was negative. This section, highlighting pontocerebellar structures demonstrates cerebellar hypoplasia and apparent claval hypertrophy.
**1f** Section of midline sagittal T1 MRI brain scan of a 9 year old male, at the level of the cerebral aqueduct, with Wolffram Syndrome, secondary to a mutation in the *WFS1* gene. The section highlighting pontocerebellar structures shows cerebellar hypoplasia and apparent claval hypertrophy.
**1g** Section of midline sagittal T1 Brain MRI of a 10 year old female with hemiplegic migraine and pathogenic *CACNA1A* mutation, demonstrating cerebellar atrophy and apparent claval hypertrophy.
**1h** Section of midline sagittal T1 Brain MRI of a 20 month old male with alpha-dystroglycanopathy. The section highlighting pontocerebellar structures demonstrates cerebellar hypoplasia and apparent claval hypertrophy.
**1i** Section of midline sagittal T1 MRI brain scan of a 6 month old female with pontocerebellar hypoplasia type 6 with pathogenic mutations in the *RARS2* gene. The imaging demonstrates cerebellar hypoplasia and apparent claval hypertrophy.

Supplementary data to this article can be found online at http://dx.doi.org/10.1016/j.ymgme.2014.03.008.

## Figures and Tables

Symbol key for Fig. 1	
	Thin vertically orientated smooth splenium of the corpus callosum
	Cerebellar atrophy with high signal consistent with gliosis
	Apparent claval hypertrophy
	Shallow optic chiasm
	Iron deposition in the globus pallidus

**Table 1:** Clinical features.

Case	1	2	3	4	5
Current age	6 yrs	8 yrs	7 yrs	30mo	17 yrs
Age at onset	8 months (developmental arrest)	12 months (developmental arrest)	18 months (developmental arrest)	22 months (developmental regression)	36 months (ataxia)
Axial hypotonia	Yes	Yes	Yes	Yes	No
Spasticity	4 limb	4 limb	4 limb	No	Yes—lower limbs
Dystonia	No	No	Yes—jaw	No	Yes—4 limb 11 years
Reflexes	Brisk initially Arreflexia- 25 months	Brisk initially Absent ankle jerks 30 months	Arreflexic	Brisk	Brisk—upper limbs Arreflexic lower limbs
Plantar responses	Extensor	Extensor	Equivocal	Extensor	Right downgoing Left equivocal
Optic atrophy	No	Yes	Yes	Yes	No
Strabismus	No	Yes	No	Yes	No
Nystagmus	No	Yes	Yes	No	No
Hypometric saccades					
Cerebellar signs	Yes	Yes	Yes	Yes	Yes
Seizures	No	No	Paroxysmal events, not confirmed as seizures	No	No
					
*Disease progression*					
Regression: *Motor*	Sitting unsupported at 8 months. Skills lost: 14 months.	Sitting unsupported at 8 months. Standing: 12 months. Skills lost: 15 months.	Sitting at 6 months, walked at 12 months. Loss of walking at 18 months. Unable to sit at 30 months. Loss of head control: 5 years.	Walking with minimal support at 15 months. Loss of supported walking: 22 months. Deterioriation in sitting posture: 24 months.	Walking at 12 months. Ataxia: 3 years. Acquisition of rollator: 14 years.
Regression: *Speech*	Loss of vocabulary: 18 months.	Loss of vocabulary: 15 months.	Loss of vocabulary: 20 months.	Loss of vocabulary: 22 months.	Intermittent dysarthria: 14 years.
Ambulation	Never achieved	Never achieved	Lost at 18 months	Lost at 24 months	Acquired rollator: 14 years
Bulbar dysfunction: *Age at onset*	14 months: Feeding difficulties and drooling. 20 months: Gastrostomy	18 months: Drooling. 24 months: Gastrostomy.	5 years: Drooling, choking, and modified diet. 7 years: Gastrostomy.	23 months: Drooling, choking on feeds, and prolonged feeding time.	16 years: Choking, prolonged feeding time.
Memory impairment: *Age at onset*					14 years: Short term memory difficulties.

**Table 2:** Investigation findings.

Case	MRI^a^ Cerebellar atrophy	MRI T2 Cerebellar high signal consistent with gliosis	MRI Hypoplastic optic chiasm and tracts	MRI Iron depositon	MRI Abnormal splenium of corpus callosum	MRI Claval hypertrophy	VERs^a^	EEG^a^	EMG^a^	NCS^a^	Biopsy
Typical findings in INAD	Almost universal	Common	Common	Late sign—40–50% cases	Vertically orientated	Undetermined	↓ or absent	High amplitude fast activity	Denervation	Distal axonal sensorimotor neuropathy	Nerve— axonal swelling and spheroid bodies
1	Yes Progressive	No	Yes	No	Yes	Yes	Nil	Abnormal^b^	Denervation	Axonal sensorimotor neuropathy	Nil
2	Yes	No	Yes	Yes—globus pallidus	Yes	Yes	Nil	Fast β2 activity	Denervation	Axonal sensorimotor Neuropathy	Nil
3	Yes	No	Yes	No	Yes	Yes	Nil	Fast β2 activity	Nil	Nil	Nil
4	Yes Progressive	Yes	Yes	No	Yes	Yes	↓	High amplitude fast activity	Denervation	Normal	Skin Normal
Typical findings in atypical NAD	Yes	Yes	No	Universal	Thin, vertically orientated and smooth	Undetermined	Normal	Normal Initially	Normal	Normal	Nerve— axonal swelling and spheroid bodies
5	Yes Progressive	No	No	Yes Basal Ganglia	Yes	Yes	Nil	Nil	Denervation	Distal axonal sensorimotor neuropathy	Muscle biopsy— neurogenic changes Respiratory chain enzymes— ↓Cytochrome oxidase—0.007 (0.014–0.034)

^a^ MRI: magnetic resonance imaging. VERs: visual evoked responses. EEG: electroencephalogram. EMG: electromyogram. NCS: nerve conduction studies. ^b^Background slowing with paroxysms of bi-posterior sharp theta and spike-slow wave activity, in wakefulness.

**Table 3:** Mutational analysis.

Case	Ethnicity	Consanguinity	Homozygous/heterozygous	Mutation type	DNA variant	Protein change
1	White Caucasian	No	Compound heterozygote	Duplication/Frameshift Missense	c.1524dupC c.1798C > T	p.Lys509Glnfs*5 p.Arg600Trp
2	White Caucasian	Yes	Homozygous	Missense	c.1756G > A	p.Gly586Arg
3	Pakistani	Yes	Homozygous	Missense	c.2375A > C	p.His792Pro
4	White Caucasian	No	Compound heterozygote	Deletion/Frameshift Duplication	c.1674delG Duplication of exons 4–7	p.Leu560Trpfs*5 unknown
5	White Caucasian	No	Compound heterozygote	Nonsense Missense	c.2370T > G c.691G > C	p.Tyr790* P.Gly231Arg
