# Supplementary figures and images for: PLA2G6-associated neurodegeneration (PLAN): Further expansion of the clinical, radiological and mutation spectrum associated with infantile and atypical childhood-onset disease
Source: Mol Genet Metab. 2014 Jun;112(2):183–9. doi: 10.1016/j.ymgme.2014.03.008 (PMC4048546; doi:10.1016/j.ymgme.2014.03.008)

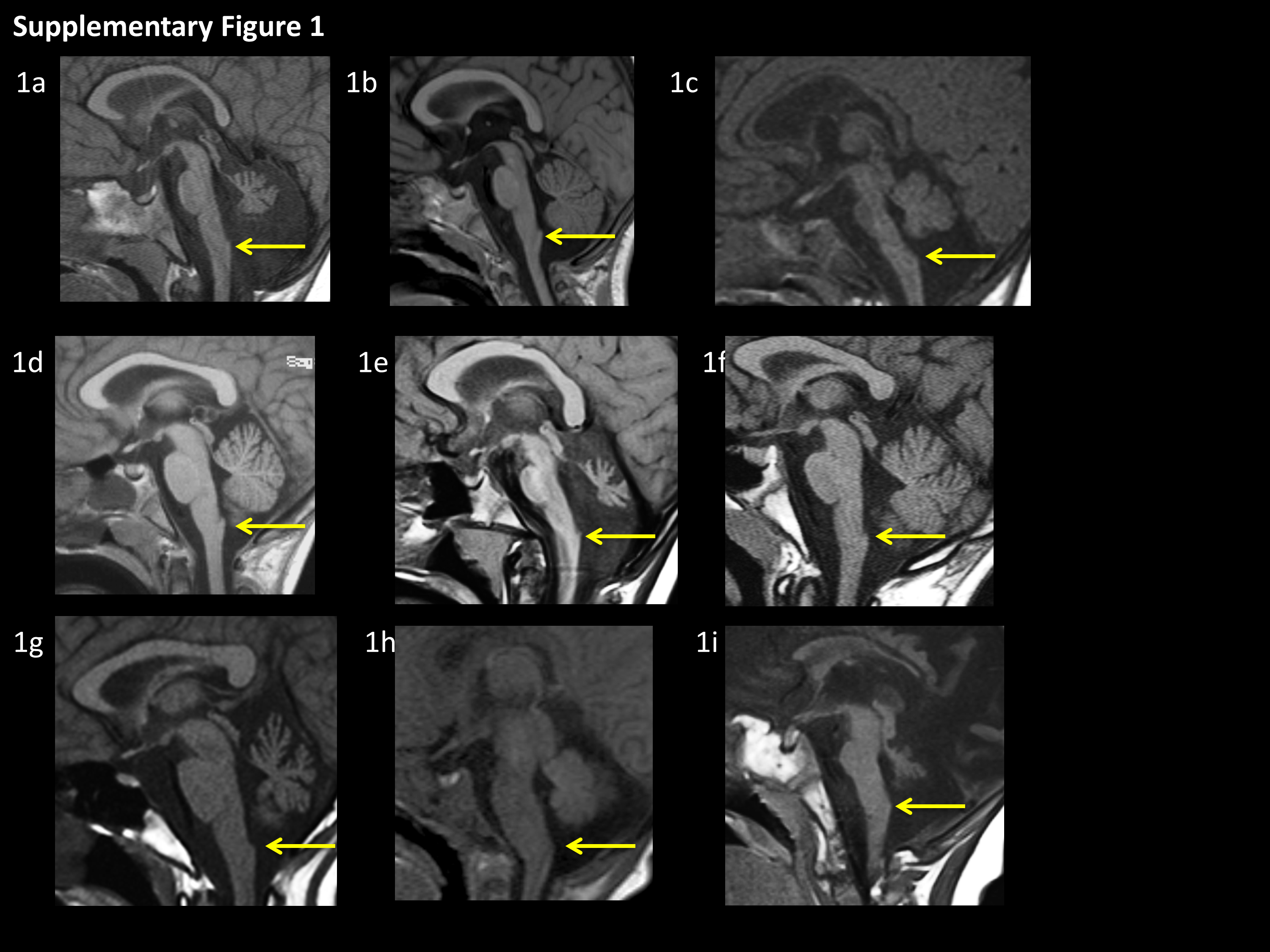

Supplement: Supplementary Fig. 1 — 9 images of midline sagittal T1 MRI brain imaging, indicating the presence of apparent claval hypertrophy (indicated by yellow arrow) in all cases. 1a Section of midline sagittal T1 MRI brain scan, at the level of the cerebral aqueduct, of a child aged 2 years 1 month with CASK mutation, cerebellar hypoplasia and apparent claval hypertrophy. 1b Section of midline sagittal T1 MRI brain scan at the level of the cerebral aqueduct, of a female aged 3 years 4 months, with pontocerebellar hypoplasia of undetermined cause. The section shows apparent claval hypertrophy and cerebellar hypoplasia. 1c Section of midline sagittal T1 MRI brain scan, at the level of the cerebral aqueduct, of a female aged 3 years and 10 months, with pontocerebellar hypoplasia type 2 secondary to TSEN54 mutation. The section capturing pontocerebellar structures, demonstrates cerebellar hypoplasia and apparent claval hypertrophy. 1d Section of midline sagittal T1 MRI brain scan, at the level of the cerebral aqueduct of a 9 years 7 month year old male, with epilepsy, learning and behavioural difficulties of undetermined cause. The section, highlighting pontocerebellar structures demonstrates cerebellar hypoplasia and apparent claval hypertrophy. 1e Section of midline sagittal T1 MRI brain scan of a 6 years and 10 month old male with a four limb movement disorder, epilepsy, microcephaly and learning difficulties of undetermined cause. PLA2G6 mutation testing was negative. This section, highlighting pontocerebellar structures demonstrates cerebellar hypoplasia and apparent claval hypertrophy. 1f Section of midline sagittal T1 MRI brain scan of a 9 year old male, at the level of the cerebral aqueduct, with Wolffram Syndrome, secondary to a mutation in the WFS1 gene. The section highlighting pontocerebellar structures shows cerebellar hypoplasia and apparent claval hypertrophy. 1g Section of midline sagittal T1 Brain MRI of a 10 year old female with hemiplegic migraine and pathogenic CACNA1A mutation, d [file mmc1.zip › Supplementary Figure 1 TIFF.tif]
